# Primary Hypoalphalipoproteinemia With Significant Premature Atherosclerosis

**DOI:** 10.1016/j.jaccas.2024.102716

**Published:** 2024-12-04

**Authors:** Sumer Moussa, Jordan Price, Jesse Frye, On Chen, Tahmid Rahman

**Affiliations:** a Department of Medicine, Stony Brook University Hospital, Stony Brook, New York, USA; b Cardiology Division, Department of Medicine, Stony Brook University Hospital, Stony Brook, New York, USA

**Keywords:** HDL-C, hypoalphalipoproteinemia, premature CAD, Tangier disease

## Abstract

Primary hypoalphalipoproteinemia is typically caused by genetic disorders and is characterized by low high-density-lipoprotein cholesterol (HDL-C). Low HDL-C has been proposed to confer an increased risk of atherosclerotic cardiovascular disease; however, a causal relationship has not been determined. We describe the case of an otherwise healthy and asymptomatic 37-year-old woman with severely low HDL-C who was found to have significant coronary artery disease in whom genetic testing supported a diagnosis of Tangier disease. Current lipid management guidelines focus on optimization of total cholesterol and low-density-lipoprotein cholesterol (LDL-C), although the lipid profile of patients with primary hypoalphalipoproteinemia typically portrays favorable non-HDL levels. Clinical trials investigating medications that target low HDL-C have failed to show a clear benefit in cardiovascular outcomes. Based on current evidence, patients with genetic disorders that manifest through low HDL-C and optimal LDL-C should be managed with lifestyle modification and statin therapy.

## Graphical Abstract

**Figure:**
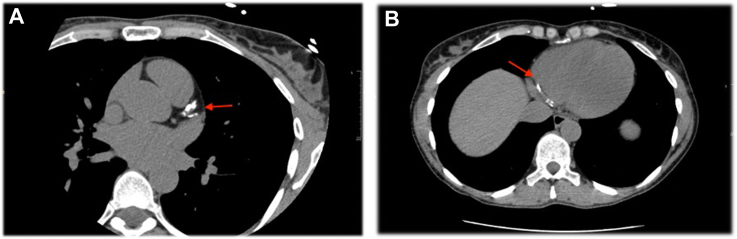

## History of Presentation

A 37-year-old woman was referred for cardiology evaluation of low high-density-lipoprotein cholesterol (HDL-C). The patient was asymptomatic, and a complete review of systems was unremarkable. Physical examination revealed a body mass index of 21 kg/m^2^, well-controlled blood pressure (118/76 mm Hg), a heart rate of 72 beats/min, and a normal cardiovascular examination. There were no corneal arcus or opacities, evidence of “fisheye” appearance, xanthomas, or mucosal or tonsillar lesions.

**Take-Home Messages**

• Genetic disorders affecting HDL-C metabolism cause primary hypoalphalipoproteinemia and may predispose to increased risk of cardiovascular disease.
• Evidence is lacking to support the use of therapeutic interventions to increase HDL-C levels for secondary prevention of coronary arterial disease.

## Past Medical History

Her medical history consisted of mitral valve prolapse, Gilbert syndrome, hypertension, and HELLP (hemolysis, elevated liver enzymes, low platelet count) syndrome. Her medications included labetalol for hypertension and an oral contraceptive (progesterone). The patient was physically active with no history of smoking, diabetes, or other cardiovascular risk factors. She had no history of menstrual irregularity or polycystic ovarian syndrome. Family history was notable for low HDL-C in the patient’s father (37 mg/dL) and sister (<20 mg/dL). The patient’s father was diagnosed with coronary artery disease (CAD) at age 65 years. There was no known family history of premature CAD.

## Differential Diagnosis

Low HDL-C is caused by both primary and secondary etiologies. Acquired causes are factors or conditions that indirectly affect HDL-C levels and include hyperparaproteinemia, severe hypertriglyceridemia, chronic inflammatory conditions, critical illness, and liver disease.^1^ Primary etiologies consist of genetic mutations that impact key steps in HDL metabolism leading to disorders including Tangier disease, familial hypoalphalipoproteinemia, and familial lecithin cholesteryl ester acyltransferase deficiency.

## Investigations

The initial lipid panel revealed a total cholesterol of 47 mg/dL, HDL-C of 5 mg/dL, low-density-lipoprotein cholesterol (LDL-C) of 24 mg/dL, triglycerides of 80 mg/dL, and very LDL of 18 mg/dL. Further laboratory testing showed a low apoA-1 of 24 mg/dL, apolipoprotein B of 52 mg/dL, lipoprotein (a) of 10.6 mg/dL, and high-sensitivity C-reactive protein of 0.24 mg/L. Liver and thyroid function tests, complete blood count, total serum protein, and basic metabolic profile were normal. There were no significant vitamin deficiencies. Genetic testing revealed 2 variants of undetermined significance to the adenosine triphosphate binding cassette transporter gene (*ABCA1*) (ABCA1, exon 20, c.2879T>C [p.Leu960Pro] heterozygous, American College of Medical Genomics and Genetics classification: Uncertain Significance; ABCA1, exon 25, c.3626C>T [p.Pro1209Leu], heterozygous, American College of Medical Genomics and Genetics classification: Uncertain Significance).

**Figure 1:**
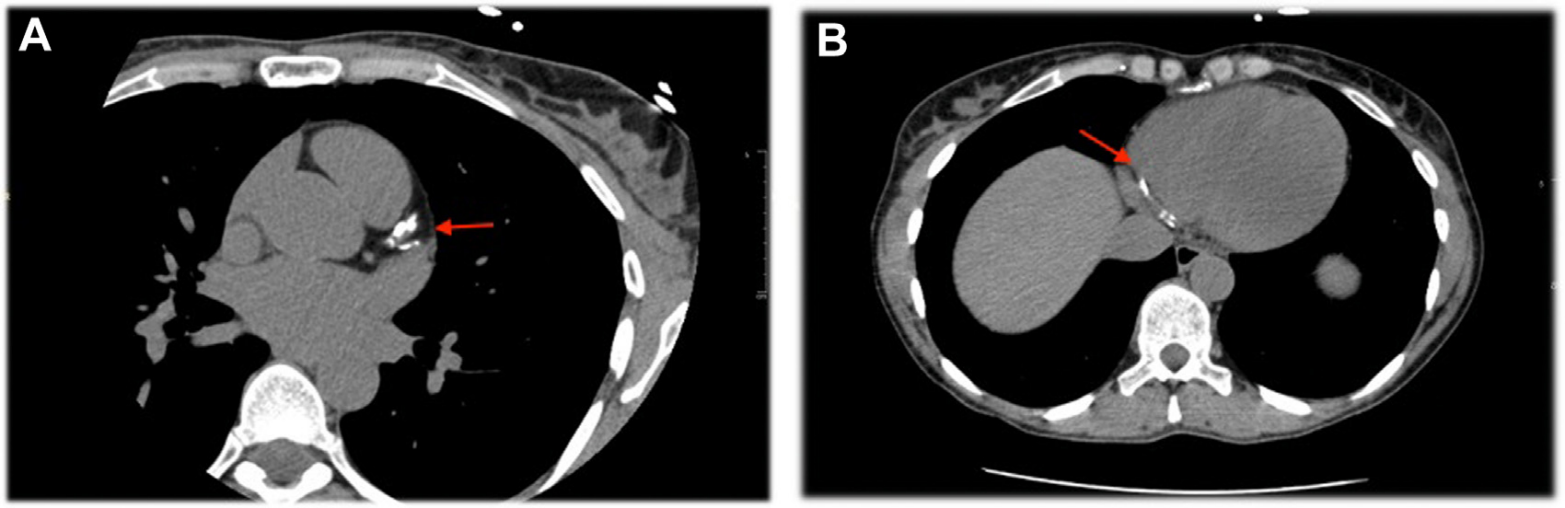
Coronary Artery Computed Tomography Coronary artery computed tomography demonstrating (A) coronary calcification of the left anterior descending artery and left circumflex artery (red arrow) and (B) coronary calcification of the right coronary artery (red arrow).

**Figure 2:**
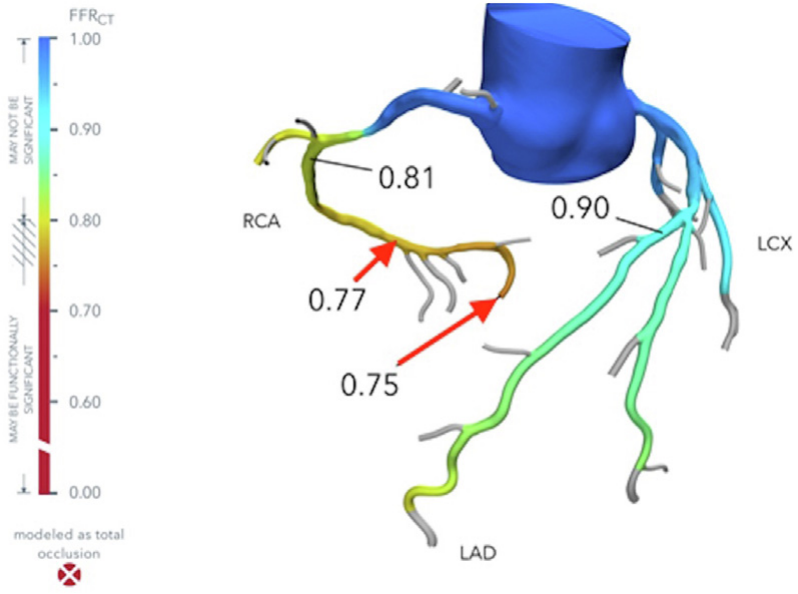
Computed Tomography Fractional Flow Reserve A CT-FFR demonstrating borderline significant stenosis of the RCA (red arrow). FFR_CT_ = computed tomography fractional flow reserve; RCA = right coronary artery

A coronary artery computed tomography was notable for borderline obstructive areas of eccentric calcified plaque in the mid to distal left anterior descending artery with 40% to 50% luminal diameter narrowing, nonobstructive eccentric calcified plaque in the proximal left circumflex artery with <40% luminal diameter narrowing, and obstructive plaque in the mid right coronary artery (RCA) with >50% luminal diameter narrowing, culminating in a whole-heart Agatston score of 981 (left anterior descending artery: 504; left circumflex artery: 15; RCA: 462) (Figure 1). Fractional flow reserve (FFR) was applied to the coronary artery computed tomography. A modeled stenosis in the mid RCA demonstrated a normal FFR measurement of 0.81 and was notable for abrupt relative change across the lesion. Additional findings included a borderline abnormal FFR value of 0.80 measured in the distal left anterior descending artery and modeled stenoses in the distal RCA and proximal right posterior lateral branch that was significant for FFR values of 0.77 and 0.75, respectively (Figure 2).

**Figure 3:**
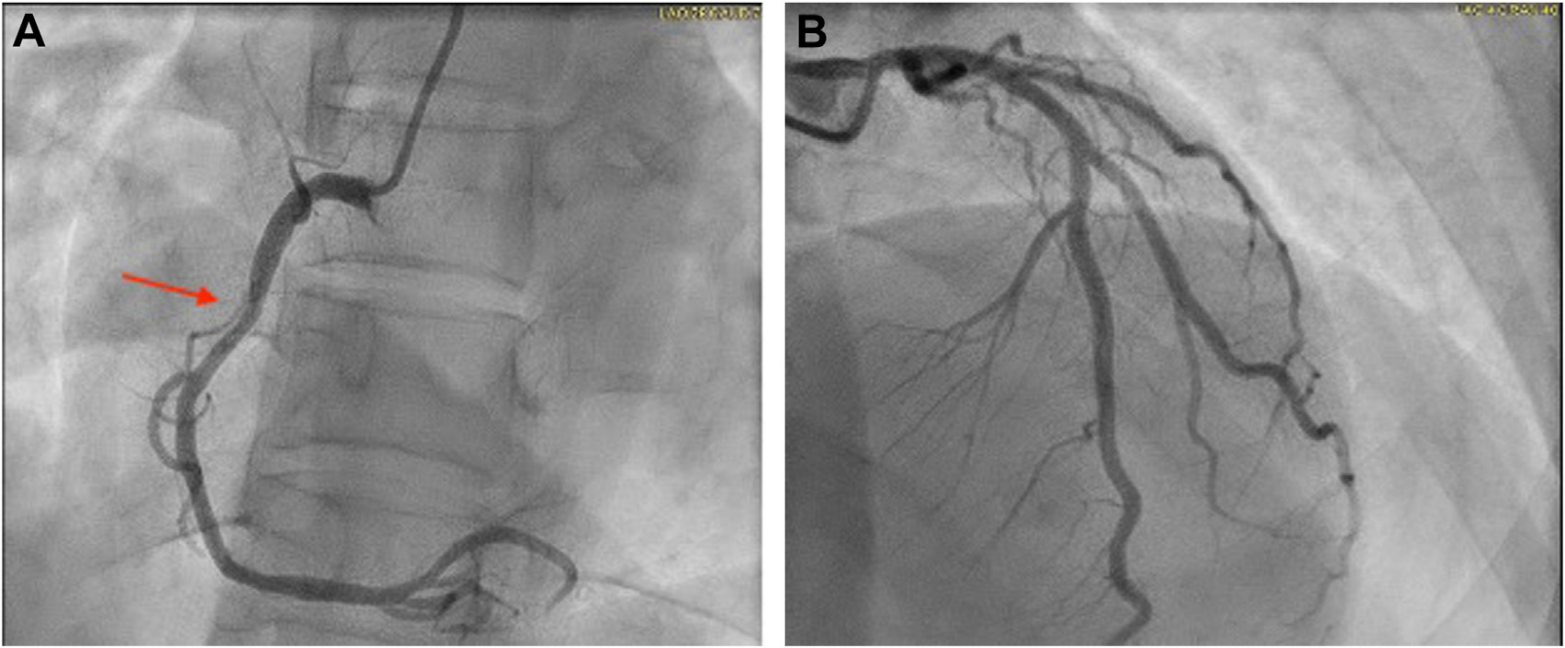
Coronary Angiography Coronary angiography demonstrating (A) nonobstructive disease of the proximal right coronary artery (red arrow) and (B) nonobstructive disease of the left anterior descending artery and left circumflex artery.

The patient subsequently underwent invasive coronary angiography that revealed nonobstructive CAD, including 50% stenosis of the mid RCA, 40% stenosis of the mid left anterior descending artery, and 30% stenosis of the ostial left circumflex artery (Figure 3). Additional diagnostic testing included lower extremity arterial duplex and magnetic resonance angiography of the brain, which were negative for vascular lesions.

## Management and Follow-Up

With persistent severely reduced HDL-C levels, a family history of low HDL-C, and *ABCA1* mutations on genetic testing of the patient and parents, the patient’s presentation was most consistent with Tangier disease. Given the significant CAD demonstrated by coronary angiography, the patient was initiated on rosuvastatin 20 mg daily and aspirin 81 mg daily. A fasting lipid profile at the 2-month interval from initiation of statin therapy showed a reduction of total cholesterol and LDL-C to 26 mg/dL and 5 mg/dL, respectively; HDL-C remained at 5 mg/dL.

## Discussion

Low HDL-C without evidence of secondary causes should raise clinical suspicion of an inherited disorder of cholesterol metabolism and prompt further investigation through genetic testing. HDL-C levels <40 mg/dL for men and 50 mg/dL for women are widely accepted as low. Severely low levels of HDL-C (<20 mg/dL) are mostly associated with mutations to the *ABCA1* gene,^1^ which may be associated with increased atherosclerotic cardiovascular disease risk.^1^^,^^2^ Two predominant genetic disorders are associated with *ABCA1* mutations. Tangier disease is inherited through an autosomal codominant pattern and is characterized by severely low HDL-C. Affected individuals may demonstrate clinical signs of impaired lipid metabolism such as orange-colored tonsils, xanthomas, xanthelasma, and arcus senilis. Heterozygous carriers often have low to normal HDL-C levels and may not exhibit any phenotypical findings. Familial hypoalphalipoproteinemia is another autosomal codominant condition that manifests as severely low HDL-C with typically normal triglycerides and LDL-C but is more commonly caused by biallelic variants in the *APOA1* gene.

The patient’s genetic testing was notable for a variant of undetermined significance of 2 alleles of *ABCA1* and no mutations to *APOA1*. The first variant described, ABCA1 c.2879T>C (p. Leu960Pro), is absent from gnomAD, whereas the second variant, ABCA1 c.3626C>T (p.Pro1209Leu), is found in 1 in 1.6 million alleles (1 in 4500 East Asian alleles). The former has a REVEL score of 0.749 and the latter is 0.876, reflecting a high likelihood that the variants are disease-causing. Genetic testing of the patient’s father and mother each demonstrated 1 heterozygous variant of undetermined significance at *ABCA1*, which corresponded to those identified in the patient (c.2879T>C, paternal; c.3626C>T, maternal) (Table 1). The lack of phenotypic findings in our patient may be explained by the considerable phenotypic heterogeneity intrinsic to Tangier disease.

**Table 1:** Lipid Profile of Patient and First-Degree Relatives

	Patient	Father	Mother	Sister
Genetic testing	VUS (2) at *ABCA1*	VUS at *ABCA1* [c.2879T>C]	VUS at *ABCA1* [c.3626C>T]	—
Apolipoprotein A-1, mg/dL	24	—	—	—
High-density-lipoprotein cholesterol, mg/dL	5	37	65	<20
Age at diagnosis of coronary artery disease, y	37	65	—	—

(—) = unable to be obtained; VUS = variant of undetermined significance.

*ABCA1* encodes the adenosine triphosphate binding cassette transporter A1 (ABCA1), which plays a vital role in cholesterol metabolism. ABCA1 is a transmembrane protein found in macrophages and other cells within peripheral tissues and is directly responsible for intracellular cholesterol efflux across the cellular membrane.^3^^,^^4^ The binding of apolipoproteins, predominantly apolipoprotein A-I, to ABCA1 leads to the outflow of intracellular cholesterol and formation of nascent HDL-C and is the first step of the reverse cholesterol transport pathway.^3^ HDL-bound cholesterol has several outcomes including esterification by lecithin-cholesterol acyl transferase and direct transport to the liver via HDL-C binding to scavenger receptor B1 on hepatocytes. The deposition of modified HDL-bound cholesterol into LDLs and very LDLs is facilitated by cholesteryl ester transfer protein.

The atheroprotective effects of HDL led to the proposal of an inverse relationship between HDL-C and cardiovascular risk. This has been well documented in population-based observational studies, including the Framingham Heart Study.^5^ Low HDL-C is also an independent risk factor associated with metabolic syndrome.^6^ Whether a causal effect exists remains debatable. Current guidelines on lipid management and atherosclerotic cardiovascular disease prevention focus on optimization of LDL-C. Several drug classes at the clinician’s disposal have shown reliable efficacy in reducing LDL-C levels in addition to conferring clinical benefit, including statins, ezetimibe, and proprotein convertase subtilisin/kexin type 9 inhibitors.^7^

Cases of low HDL-C and normal or low LDL-C, as seen in primary hypoalphalipoproteinemia, present a unique challenge in the choice of treatment. Several trials investigating pharmacologic interventions that aim to increase HDL-C have not shown clinical benefit of atherosclerotic cardiovascular disease–related outcomes. In the AIM-HIGH trial, the addition of niacin to statin therapy in patients with established cardiovascular disease and low HDL-C levels at baseline resulted in no significant reduction of cardiovascular events despite significant increases in HDL-C levels.^8^ The HPS2-THRIVE study also investigated the role of niacin in addition to statin therapy in patients with established atherosclerotic disease. Although there was an observed increase of HDL-C in the treatment arm, a significant reduction of major vascular outcomes was not seen.^9^ Schwartz et al^10^ investigated the effects of the cholesteryl ester transfer protein inhibitor dalcetrapib in the dal-OUTCOMES trial. In patients with recent acute coronary syndrome, treatment with dalcetrapib increased plasma HDL-C but did not reduce the risk of further cardiovascular symptoms when compared with standard therapy.^10^

It is believed that low LDL-C in patients with Tangier disease is protective against premature CAD. However, in the absence of biochemical or other established cardiovascular risk factors, the patient’s isolated low HDL-C is the most likely cause of significant premature CAD.

## Conclusions

Patients with primary hypoalphalipoproteinemia exhibit severely low HDL-C and may be at increased risk for atherosclerotic cardiovascular disease. Genetic testing should be performed in individuals in whom primary hypoalphalipoproteinemia is suspected. Clinical trials investigating the role of therapeutic interventions aimed at increasing HDL-C have yet to show benefit in reducing risk of cardiovascular events. Based on current literature and guidelines, the management of primary hypoalphalipoproteinemia should focus on lifestyle modifications including smoking prevention, blood pressure control, and LDL-C optimization. Further studies are needed to investigate possible therapeutic targets in patients with genetic causes of altered lipid metabolism.

**Table:** Visual Summary: Timeline of the Case

Steps	Events
1.	Patient was referred to the cardiology clinic for evaluation of low HDL-C. She was asymptomatic with an unremarkable physical examination. Further laboratory testing was ordered.
2.	Laboratory values were as follows: Total cholesterol: 47 mg/dL HDL-C: 5 mg/dL LDL-C: 24 mg/dL Triglycerides: 80 mg/dL Very LDL: 18 mg/dL Apolipoprotein B: 52 mg/dL Lipoprotein a: 10.6 mg/dL High-sensitivity C-reactive protein: 0.24 mg/L Thyroid function tests, liver function tests, and complete blood count were normal. Genetic testing was as follows: 2 variants of undetermined significance to the *ABCA1* gene • ABCA1 c.2879T>C (p.Leu960Pro): paternally inherited • ABCA1 c.3626C>T (p.Pro1209Leu): maternally inherited
3.	Imaging and diagnostic testing were as follows: Coronary artery computed tomography: Agatston score of 981 (LAD 504, LCx 15, RCA 462) Mid to distal LAD and proximal LCx with nonobstructive calcified plaque Mid RCA with obstructive calcified plaque Coronary angiography was as follows: Nonobstructive coronary artery disease, including mid RCA 50% stenosis, mid LAD 40% stenosis, and ostial LCx 30% stenosis
4.	Patient was started on statin and aspirin for secondary prevention of coronary artery disease in addition further lifestyle modification. A repeat lipid profile at a 2-month interval from initiation of therapy showed reduction of total cholesterol and LDL-C to 26 mg/dL and 5 mg/dL respectively. HDL-C remained at 5 mg/dL.

HDL-C = high-density-lipoprotein cholesterol; LAD = left anterior descending artery; LCx = left circumflex artery; LDL-C = low-density-lipoprotein cholesterol; RCA = right coronary artery.

## Funding Support and Author Disclosures

Dr Chen is a speaker for Amgen and Zoll. Dr Rahman is on the advisory board for Lp(a) in South Asians for Novartis. All other authors have reported that they have no relationships relevant to the contents of this paper to disclose.
